# Expulsion of the Entire Ovum at the Full Time

**Published:** 1848-01

**Authors:** 


### EDINBURGH OBSTETRICAL SOCIETY.

*Expulsion of the entire ovum at the full time.*—Dr. M. Barry read the following case: Mrs. M., aged 22, Dyer's Close, Cowgate, gave birth to her second child on the 2d of February, 1846. States that the last catamenia began and terminated at the end of the preceding April.

The medical attendant on arriving found the first stage of labour complete; the presenting parts being the nates and right foot, in a position corresponding to the third position of the head—i. e., with the sacrum of the child directed towards the right sacro-iliac synchondrosis of the mother. "Pains" returned every three or four minutes, and the passages were not only well lubricated, but apparently very ample. With a breech presentation, however, it was not anticipated that the labour would be immediately at an end, and still less that it would be completed in the following remarkable manner.

The patient having expressed a wish to obey a call of nature, the medical attendant withdrew; but before many minutes had elapsed, he was urgently requested to return, and found the child not only born, but lying with the liquor amnii in its unruptured bag of membranes, and the placenta expelled along with it; the whole having been precipitated almost without a "pain" into the pôt de chambre. The membranes were ruptured without delay; after which a tap on the nates and the dashing of a few drops of cold water on the chest were found sufficient to establish the free respiration of the child.

The mother is a very little woman. Some flooding followed. Forty hours after the birth, the child weighed 5 lb. 3 oz. 3 drachms. When born it must have weighed more, for it had not received nourishment nearly equal in quantity to the evacuated meconium.

Various similar cases were mentioned by other members.

